# Supplementary material for: A pan-cancer analysis of homeobox family: expression characteristics and latent significance in prognosis and immune microenvironment
Source: Front Oncol. 2025 Feb 6;15:1521652. doi: 10.3389/fonc.2025.1521652 (PMC11840236; doi:10.3389/fonc.2025.1521652)
Supplement: Supplementary Figure 1 — Mutations of each HOX gene in 32 cancers (TCGA, PanCancer Atlas) by cBioportal. [file DataSheet1.zip › Suppl.files/raw code.zip!/code/4.survival/survivalplot.zip!/survivalplot/HOXC6_OS.LIHC.pdf]

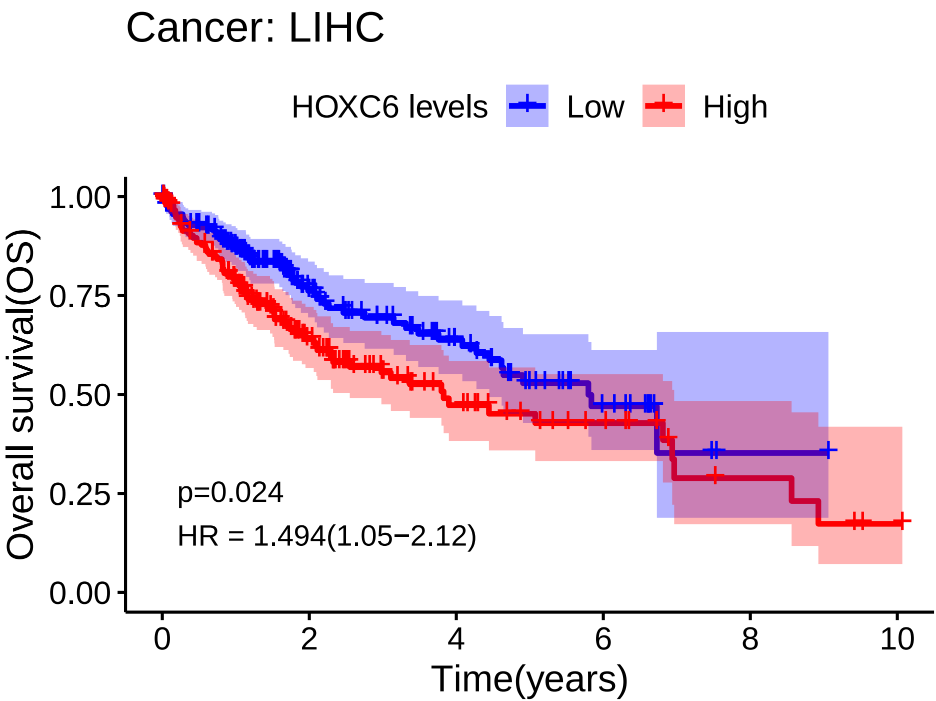


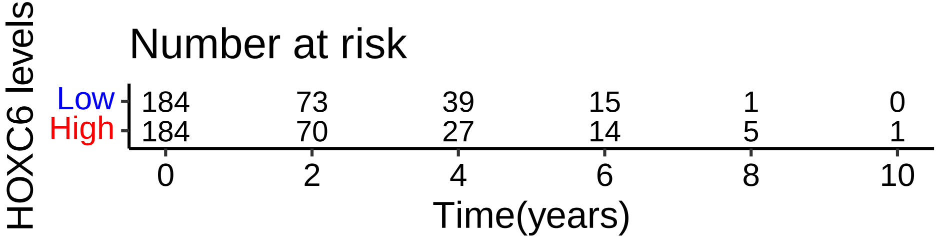


| HOXC6 levels | 0 | 2 | 4 | 6 | 8 | 10 |
|---|---|---|---|---|---|---|
| Low | 184 | 73 | 39 | 15 | 1 | 0 |
| High | 184 | 70 | 27 | 14 | 5 | 1 |

Time(years)
